# Ophthalmic involvement in VEXAS syndrome and its influence on mortality: insights from the international AIDA network registry

**DOI:** 10.3389/fimmu.2026.1709085

**Published:** 2026-06-19

**Authors:** Jurgen Sota, Andrea Hinojosa-Azaola, Eduardo Martín-Nares, Paolo Sfriso, Sara Bindoli, Pravin Hissaria, Mark Beecher, José Hernández-Rodríguez, Verónica Gómez-Caverzaschi, Micol Frassi, Francesca Crisafulli, Lorenzo Dagna, Corrado Campochiaro, Serena Bugatti, Alessandra Milanesi, Guillermo Ruiz-Irastorza, Adriana Soto-Peleteiro, Matteo Piga, Ombretta Viapiana, Abdurrahman Tufan, Ertugrul Cagri Bolek, Paola Triggianese, Marcella Prete, Jessica Sblachiero, Valeria Caggiano, Antonio Vitale, Henrique Ayres Mayrink Giardini, Giuseppe Lopalco, Fabrizio Conti, Paolo Moscato, Chiara Cardamone, Ewa Wiesik-Szewczy, Andrés González-García, Amato De Paulis, Rosetta Vitetta, Perla Ayumi Kawakami-Campos, Alessandra Brancaleoni, Andrea Mercanti, Alessandra Renieri, Monica Bocchia, Gaafar Ragab, Vishali Gupta, Alejandra de-la-Torre, Bruno Frediani, Carmelo Gurnari, Luca Cantarini, Claudia Fabiani

**Affiliations:** 1 Rheumatology Unit, Department of Medical Sciences, Surgery and Neurosciences, University of Siena and Azienda Ospedaliero-Universitaria Senese [European Reference Network (ERN) for Rare Immunodeficiency, Autoinflammatory, and Autoimmune Diseases (RITA) Center], Siena, Italy; 2 Department of Immunology and Rheumatology, Instituto Nacional de Ciencias Médicas y Nutrición Salvador Zubirán, Mexico City, Mexico; 3 Rheumatology Unit, Department of Medicine, University of Padua, Padua, Italy; 4 Department of Clinical Immunology and Allergy, Royal Adelaide Hospital, Adelaide, SA, Australia; 5 Department of Immunopathology, SA Pathology, Adelaide, SA, Australia; 6 Autoinflammatory Diseases Clinical Unit, Department of Autoimmune Diseases, Hospital Clinic of Barcelona, Institut d’Investigacions Biomèdiques August Pi i Sunyer (IDIBAPS), University of Barcelona, Center of the European Reference Network (ERN) for Rare Immunodeficiency, Autoinflammatory and Autoimmune Diseases (RITA), Barcelona, Spain; 7 Rheumatology and Clinical Immunology, Spedali Civili and Department of Clinical and Experimental Sciences, European Reference Network (ERN) for Rare Immunodeficiency, Autoinflammatory and Autoimmune Diseases (RITA) Center, University of Brescia, Brescia, Italy; 8 Division of Immunology, Transplants and Infectious Diseases, Università Vita-Salute San Raffaele, Milan, Italy; 9 Unit of Immunology, Rheumatology, Allergy and Rare Diseases, Istituto di Ricovero e Cura a Carattere Scientifico (IRCCS) Ospedale San Raffaele, European Reference Network (ERN) for Rare Immunodeficiency, Autoinflammatory and Autoimmune Diseases (RITA) Center, Milan, Italy; 10 Rheumatology Department, Istituto di Ricovero e Cura a Carattere Scientifico Policlinico S. Matteo Fondazione, University of Pavia, Pavia, Italy; 11 Faculty of Medicine and Nursery, University of the Basque Country, UPV/EHU, Leioa, Biscay, Spain; 12 Autoimmune Diseases Unit, Biocruces Bizkaia Health Research Institute, Barakaldo, Biscay, Spain; 13 Rheumatology Unit, Department of Medical Sciences and Public Health, University and Azienda Opsedaliero-Universitaria (AOU) of Cagliari, Cagliari, Italy; 14 Rheumatology Unit, Department of Medicine, University and Azienda Ospedaliera Universitaria Integrata of Verona, Verona, Italy; 15 Division of Rheumatology, Department of Internal Medicine, Gazi University Hospital, Ankara, Türkiye; 16 Unità Operativa Complessa (UOC) Medicina Interna - Unità Operativa Semplice Dipartimentale (UOSD) Geriatria, Università di Roma Tor Vergata, Rome, Italy; 17 Department of Biomedicine and Prevention, University of Rome Tor Vergata, Rome, Italy; 18 Internal Medicine Unit, Department of Interdisciplinary Medicine, University of Bari “Aldo Moro”, Bari, Italy; 19 Rheumatology Division, Faculdade de Medicina, Hospital das Clinicas (HCFMUSP), Universidade de Sao Paulo, Sao Paulo, Brazil; 20 Department of Precision and Rigenerative Medicine and Ionian Area (DiMePRe-J), Polyclinic Hospital, University of Bari, Bari, Italy; 21 Rheumatology Unit, Department of Clinical Internal, Anesthesiologic and Cardiovascular Sciences, Sapienza University of Rome, Rome, Italy; 22 Department of Medicine, University Hospital San Giovanni di Dio e Ruggi d’Aragona, Salerno, Italy; 23 Department of Internal Medicine, Pneumonology, Allergology and Clinical Immunology, Central Clinical Hospital of the Ministry of National Defense, Military Institute of Medicine, National Research Institute, Warsaw, Poland; 24 Systemic Autoimmune Diseases Unit, Department of Internal Medicine, Hospital Universitario Ramón y Cajal, IRYCIS, Madrid, Spain; 25 Department of Translational Medical Sciences, Section of Clinical Immunology, University of Naples Federico II, Naples, Italy; 26 Center for Basic and Clinical Immunology Research (CISI), World Allergy Organization (WAO) Center of Excellence, University of Naples Federico II, Naples, Italy; 27 Unit of Rheumatology, Azienda Sanitaria Locale Vercelli (ASL VC) Sant’ Andrea Hospital, Vercelli, Italy; 28 Department of Ophthalmology, Instituto Nacional de Ciencias Médicas y Nutrición Salvador Zubirán, Mexico City, Mexico; 29 Department of Ophthalmology, Azienda Unità Sanitaria Locale (AUSL) Romagna - Ospedale di Rimini, Rimini, Italy; 30 Medical Genetics, Department of Medical Biotechnologies, University of Siena, Siena, Italy; 31 Department of Medical Biotechnologies, Med Biotech Hub and Competence Center, University of Siena, Siena, Italy; 32 Genetica Medica, Azienda Ospedaliero-Universitaria Senese, [European Reference Network (ERN) for Rare Immunodeficiency, Autoinflammatory and Autoimmune Diseases (RITA) Center] Siena, Siena, Italy; 33 Hematology Unit, Department of Medical Sciences, Surgery and Neurosciences, University of Siena and Azienda Ospedaliero-Universitaria Senese [European Reference Network (ERN) for Rare Immunodeficiency, Autoinflammatory and Autoimmune Diseases (RITA) Center] Siena, Siena, Italy; 34 Rheumatology and Clinical Immunology Unit, Internal Medicine Department, Faculty of Medicine, Cairo University, Giza, Egypt; 35 Faculty of Medicine, Newgiza University, 6th of October City, Egypt; 36 Advanced Eye Centre, Postgraduate Institute of Medical Education and Research, Chandigarh, India; 37 Neuroscience Research Group (NEUROS), NeuroVitae Center, Escuela de Medicina y Ciencias de la Salud, Universidad del Rosario, Bogotá, Colombia; 38 Department of Translational Hematology and Oncology Research, Taussig Cancer Institute, Cleveland Clinic, Cleveland, OH, United States; 39 Ophthalmology Unit, Department of Medicine, Surgery and Neurosciences, University of Siena and Azienda Ospedaliero-Universitaria Senese [European Reference Network (ERN) for Rare Immunodeficiency, Autoinflammatory, and Autoimmune Diseases (RITA) Center], Siena, Italy

**Keywords:** inflammatory orbitopathy, mortality, scleritis, uveitis, VEXAS syndrome

## Abstract

Orbital inflammation is the most common presentation of VEXAS, with any orbital structure potentially affected. Non-sight-threatening and uncomplicated anterior non-granulomatous uveitis and anterior diffuse scleritis follow in frequency, along with episcleritis. Ophthalmic involvement was significantly associated with relapsing polychondritis (*p* = 0.014), with an increased chance of a fatal outcome (RR 5.87, *p* = 0.016) and independently predicts a higher mortality rate (OR 3.72, *p* = 0.026). Treatment of ophthalmic involvement showed full or partial response to glucocorticosteroids alone in 66.7% of cases. Ophthalmic involvement is common in VEXAS syndrome and signals a poorer prognosis in terms of mortality, highlighting the need for close monitoring.

## Introduction

Vacuoles, E1 enzyme, X-linked, Autoinflammatory, Somatic (VEXAS) syndrome is a recently described life-threatening monogenic X-linked autoinflammatory disease caused by acquired mutations on the *UBA1* gene in hematopoietic progenitors. The *UBA1* mutations lead to defective ubiquitination pathways, resulting in systemic inflammatory manifestations and hematologic disorders (1, 2). Systemic inflammation in VEXAS syndrome can affect virtually any organ or tissue. Among these, the skin is the most commonly involved organ. On the other hand, the eye and periocular/orbital tissues appear to be involved in nearly half of patients during the disease course (3), and in those with eye involvement it was present in over 92% at disease onset. Although initial reports, including those from our research network, have described orbital inflammation, episcleritis, uveitis, and scleritis as ophthalmic manifestations of VEXAS (3–5), a detailed characterization of the ocular inflammation, particularly with regard to uveitis and scleritis is still lacking. Additionally, preliminary data from our research network reported a significant association between orbital involvement and relapsing polychondritis in the context of VEXAS, and noted that 5 out of 6 deaths occurred in patients who had experienced ophthalmic inflammation.

In this context, we aimed to detail the ophthalmic involvement of patients with VEXAS syndrome, including its anatomical and pathological classification. Furthermore, we sought to further assess, in a larger multicenter cohort, the role of ophthalmic involvement as a potential predictor of mortality.

## Methods

Medical records of VEXAS patients were reviewed. Data were retrieved from the international AIDA Network Registries on VEXAS syndrome, uveitis and scleritis (6–8). The study adheres to the Declaration of Helsinki and was approved by the University of Siena Local Ethics Committee (Ref. No. 14951). All patients provided written consent.

The primary aim of the study was to detail ophthalmic involvement and assess its association with disease outcome, expressed in mortality. Secondary objectives included the evaluation of (i) the potential association between ophthalmic inflammation and relapsing polychondritis, and (ii) the response of ophthalmic inflammation to local and systemic treatments.Data were analyzed using SPSS (IBM Corp., Armonk, NY, US). Normality was assessed with the Shapiro-Wilk test. Quantitative variables were expressed as mean ± SD or median (IQR), and qualitative as frequencies (%). Pearson’s Chi-Square test was employed. Predictors of fatal events were analyzed through binary logistic regression analysis with the stepwise backward method. Two variables were included in the model: ocular involvement and the presence/absence of an associated systemic disease. All *p*-values were two-sided, with statistical significance set at 0.05.

## Results

**Figure 1:**
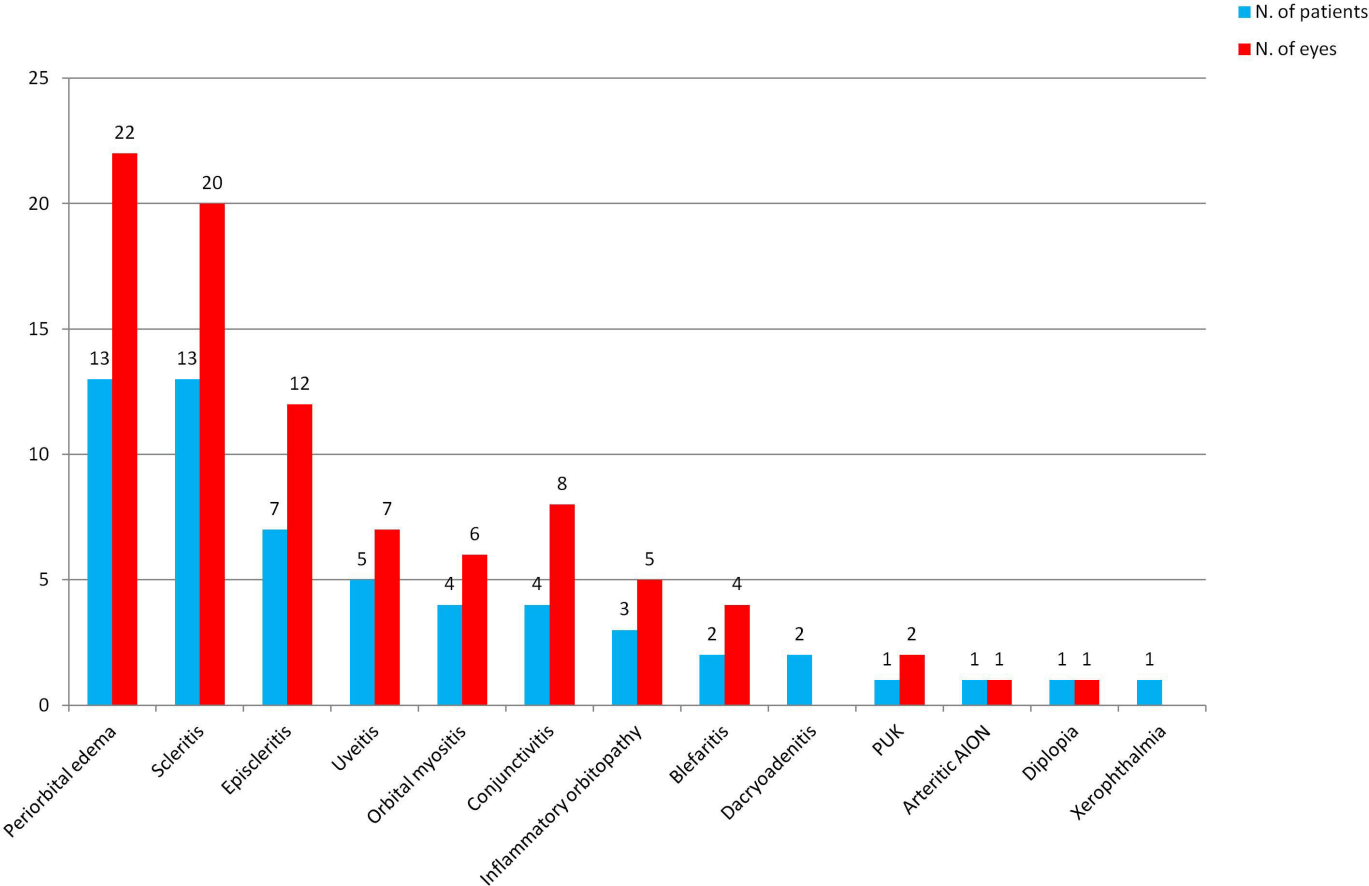
Bar chart illustrates a detailed description of the ocular findings in our cohort. *AION* Anterior ischemic optic neuropathy, *PUK* peripheral ulcerative keratitis.

A total of 86 patients (83 males, 3 females) with genetically confirmed VEXAS syndrome were enrolled. Ophthalmic inflammation occurred in 41 cases (47.7%), present at disease onset in 26/35 (74.3%) and before diagnosis in 33/35 (94.3%). Onset age was missing in 6. **Figure 1** illustrates all types of ophthalmic involvement encountered in our cohort.

Regarding non-infectious uveitis and scleritis, the most frequent anatomical pattern was anterior uveitis and anterior diffuse scleritis, respectively. No complications related to uveitis or scleritis were observed. **Table 1** lists the main demographic, clinical and therapeutic characteristics of the entire cohort.

**Table 1:** Demographic, clinical and therapeutic findings of patients affected by VEXAS syndrome.

N° patients	86
Mean age at systemic disease onset ± SD (years)	67.13 ± 10.76
Mean age at onset of ocular involvement	67.33 ± 11.20
Mean age at VEXAS syndrome diagnosis ± SD (years)	71.02 ± 10.36
Mean ± SD disease duration at diagnosis of VEXAS syndrome (years) – median (IQR)	3.84 ± 3.45 – 3.00 (4.63)
Ethnic origin	White (n=70, 81.4%) Hispanic (n=16, 18.6%)
Genetic mutations	M41T (n=40, 46.5%) (pathogenetic) M41V (n=19, 22.1%) (pathogenetic) M41L (n=15, 17.4%) (pathogenetic) Others (n=12, 14.0%) c.118-1G>C (n=3) (pathogenetic) G477A (n=3) (likely pathogenetic) S56F (n=2) (likely pathogenetic) c.118-2A>G (n=2) (pathogenetic) unspecified (n=2)
Past advanced treatments with biologic agents and small molecules^§^	Tocilizumab (n=4), Anakinra (n=4), Etancercept (n=2), Ruxolitinib (n=2), Infliximab (n=2), Upadacitinib (n=2), Abatacept (n=1), Sarilumab (n=1)
Anatomical classification of uveitis and scleritis (n° of eyes)	AU (*n* = 4) IU (*n* = 2) AS (*n* = 14) AS + PUK (n=1) PS (*n* = 1) AU + AS (*n* = 1) Unknown (n=3)
Treatment response of scleritis	CR (n=10, 83.3%) • GC (n=7) • Topical NSAIDs (n=1) • MTX (n=1) • HCQ (n=1) PR (n=2, 16.7%) • GCs (n=2)
Treatment response of uveitis	CR (n=2, 40%) • GCs (n=2) PR (n=2, 40%) • GCs (n=2) No data (n=1, 20%)
Treatment response of inflammatory orbitopathy	CR (n=10, 100%) • GCs (n=5) • GCs + Ruxolitinib (n=1) • GCs + Canakinumab (n=1) • GCs + Tofacitinib (n=1) • GCs + Adalimumab (n=1) • GCs + Rituximab (n=1)
Associated disease manifestations	Fever (n=64, 74.4%) Skin involvement (n=75, 87.2%) Hematological manifestations (n=80, 93.0%) Lung involvement (n=24, 27.9%) Articular involvement (n=66, 76.4%) Vascular involvement (n=40, 46.5%) Neurological involvement (n=19, 22.1%) Gastrointestinal involvement (n=20, 23.3%) Urogenital system involvement (n=4, 4.7%) Relapsing polychondritis (n=23, 26.7%)
Causes of death (n° of patients)	Acute respiratory failure (n=3) Unspecified lung diseases (n=2) Infectious complications (n=2) Non-traumatic intestinal perforation (n=1) Myocardial infarction (n=1) Pulmonary heart disease (n=1) Acute pancreatitis (n=1) Vasculitis (n=1) Unreported (n=4).

*AS*, anterior scleritis; *AU*, anterior uveitis; *CR*, complete response; *GCs*, glucocorticosteroids; *HCQ*, hydroxychloroquine; *IQR*, interquartile range; *IU*, intermediate uveitis; *MTX*, methotrexate; *NSAIDs*, non-steroid anti-inflammatory drugs; *PR*, partial response; *PS*, posterior scleritis; *PUK*, peripheral ulcerative keratitis; *SD*, standard deviation.

*Standardization of Uveitis Nomenclature (SUN) uveitis classification criteria, as well as the grading and classification system for scleritis proposed by Sen et al.

^§^ Past treatments with biologics and small molecules, administered for extra-ocular manifestations.

Ophthalmic involvement was significantly associated with a higher mortality rate (RR, 5.87 C.I. 1.39-24.75, *p* = 0.016). A fatal outcome was recorded in 13 and 3 patients with and without ocular involvement, respectively. This finding was corroborated in the regression model, with ophthalmic involvement significantly predicting a higher mortality rate (OR 3.72, C.I. 1.169-11.815, *p* = 0.026). The statistical significance was maintained after the exclusion of patients showing ocular surface disorders such as blepharitis and conjunctivitis that may display a multifactorial genesis (RR 7.90, C.I. 2.27-27.52, *p* < 0.001). Fatal events are recorded in **Table 1**.

The frequency of relapsing polychondritis in VEXAS patients was significantly higher in patients with ophthalmic involvement (16/41) compared to those without (7/45) (*p* = 0.014).

Among patients with uveitis and scleritis, 9 patients (52.9%) showed complete and 4 (23.5%) partial response to topical and/or systemic glucocorticosteroids (GCs). Inflammatory orbitopathy was treated with GCs alone in 5 cases (50%), while advanced therapies (biologic agents or small molecules) were used in other 5 (50%) to achieve remission.

## Discussion

This is the first study detailing the anatomical and pathological features of ophthalmic involvement in VEXAS syndrome. In all cases, inflammation was non-infectious and typically present at onset or before diagnosis, highlighting its diagnostic value and the essential role of ophthalmologists in a multidisciplinary setting.

Ophthalmic inflammation in VEXAS syndrome is heterogeneous and nonspecific and was present in nearly half of the cohort, following hematologic (93%), skin (87.2%) and articular involvement (76.4%). Our cohort’s features are epidemiologically consistent with previous reports (3, 5, 9), with orbital inflammation being most common (50%), affecting structures anterior and posterior to the orbital septum, including extraocular muscles, orbital fat, and lacrimal gland. Uveitis was predominantly anterior and non-granulomatous, with only one case of intermediate uveitis. In no patient did inflammation or its complications affect the critical structures of the posterior segment. Scleritis was anterior and diffuse.

Uveitis and scleritis responded either completely (53%) or partially (24%) to topical and/or systemic GCs, without the association of chronic disease-modifying agents. The anatomical localization of ocular inflammation may explain the optimal response to GCs alone, as uncomplicated and non-sight-threatening anterior uveitis and anterior diffuse (non-necrotizing) scleritis might not require conventional or advanced treatments with biologic disease modifiers and small molecules. On the other hand, patients with inflammatory orbitopathy required the addition of biologic agents or small molecules in half of the cases.

Ophthalmic involvement was significantly associated with increased mortality and emerged as an independent predictor of fatal outcome, indicating the need for closer systemic monitoring and potentially more aggressive treatment in this subset of patients. In our cohort, the most frequent causes of death were lung disease, whereas other multicenter studies have reported infections and intestinal perforations as leading causes (3, 10). The mechanisms linking ophthalmic involvement to higher mortality remain unclear, and it is still unknown which specific ophthalmic manifestations directly impact prognosis—questions that warrant future investigation.

We found a statistically significant link with relapsing polychondritis (RP), confirming previous AIDA network findings (4). Thus, screening for *UBA1* mutations should be considered in elderly male patients initially diagnosed with RP and presenting ophthalmic inflammation. These findings align with a large French cohort reporting a higher frequency of ocular involvement in VEXAS-RP compared to idiopathic RP (11).

In summary, our findings support that ophthalmic inflammation is an early, common feature of VEXAS syndrome, potentially affecting any orbital structure. Uveitis typically expresses as anterior, with a non-granulomatous pattern, while scleritis presents as anterior and diffuse. Despite being generally responsive to GCs and/or DMARDs, ophthalmic involvement is linked to higher mortality and demands careful monitoring to ensure appropriate patient follow-up.

## Data Availability

Access to data registry is restricted to protect confidential or proprietary information according to the strict normes of General Data Protection Regulation (GDPR) of 2016. They are available upon reasonable request to the corresponding authors (cantariniluca@hotmail.com).
